# Permanent Complete Heart Block: A Rare Complication of Influenza Infection

**DOI:** 10.7759/cureus.51166

**Published:** 2023-12-27

**Authors:** Mohamed Badheeb, Stuart Zarich, Faria Islam Fara, Md. Mashiul Alam

**Affiliations:** 1 Internal Medicine, Yale New Haven Health, Bridgeport Hospital, Bridgeport, USA; 2 Cardiology, Yale New Haven Health, Bridgeport Hospital, Bridgeport, USA; 3 Biotechnology Program, Department of Mathematics & Natural Sciences, BRAC University, Dhaka, BGD; 4 Cardiovascular Disease, Mayo Clinic, Rochester, USA

**Keywords:** permanent av block, complete av block, bradycardia, influenza a, heart block

## Abstract

The cardiovascular complications of viral illnesses are often underestimated in clinical practice. The influenza virus, one of the most prevalent viral infections, has been associated with a wide spectrum of arrhythmias that are typically transient and self-resolving. We present the case of a 60-year-old female with no prior cardiac comorbidities who developed a complete heart block after an influenza infection. She presented to the clinic with flu-like symptoms and was found to have a complete heart block with a junctional escape rhythm. Polymerase chain reaction testing subsequently confirmed an influenza A infection. She was initially placed on a temporary pacemaker. However, a permanent dual-chamber pacemaker was implanted as bradycardia persisted. Later follow-ups in the cardiology clinic showed that the patient remained dependent on the pacemaker. While there are a few descriptions of influenza-induced transient atrioventricular block, cases of influenza-induced permanent complete heart block are extremely rare, particularly in the absence of severe myocardial inflammation.

## Introduction

The burden of the influenza virus on healthcare systems is significant. Previous reports have estimated annual deaths exceeding 300,000 patients with critical influenza infections [1]. While influenza predominantly manifests as a transient respiratory illness, it can present with notable systemic manifestations, including cardiovascular complications [2,3]. Global reports indicate that over 100,000 deaths in influenza patients were secondary to cardiovascular complications [4,5]. Furthermore, cardiovascular comorbidities secondary to the influenza virus have been shown to be more prevalent and serious compared to other viral illnesses, though they are less emphasized [6].

The influenza virus can affect both cardiac myocytes and conductive systems, with reports indicating that 10% of viral myocarditis cases are attributed to influenza [7]. Notably, a wide spectrum of myocardial dysfunction has been documented among influenza patients, varying from localized wall motion abnormalities to fulminant myocarditis with biventricular failure [8]. Likewise, influenza has been linked to various cardiac arrhythmias and conductive abnormalities. In most cases, asymptomatic sinus bradycardia and tachycardia are commonly seen. However, atrial and ventricular arrhythmias, heart block, or even sudden cardiac death have also been reported [9-11]. Reports of heart block in influenza infections have been limited to low-grade atrioventricular block, and these cases usually have concomitant severe myocardial inflammation [10-12]. Only a few case reports of complete heart block (CHB) secondary to influenza virus have been reported in the literature. Herein, we present the case of a patient presenting with CHB secondary to a mild influenza A virus infection that necessitated permanent pacemaker placement.

## Case presentation

A 60-year-old female presented to the clinic with a four-day history of sore throat, nasal congestion, and generalized fatigue. She denied having a fever, chest pain, or dyspnea. The patient had no recent history of tick bites or travel and was not on any medications, including atrioventricular nodal blocking agents. She had a past medical history of mild chronic obstructive pulmonary disease and bilateral sensorineural hearing loss. Her social history was significant for consuming three to four glasses of alcohol nightly, with episodic binge drinking, and she had quit smoking a few years before the presentation. On examination, the patient was afebrile at 98.2°F (36.8°C). Bradycardia with a heart rate of 36 beats per minute and blood pressure of 173/73 mmHg were noted, distinct from her prior documented heart rate readings in previous office visits, which ranged between 60 and 85 beats per minute. Other remarkable findings included facial plethora and pharyngeal hyperemia. The rest of her clinical examination was unremarkable.

**Figure 1:**
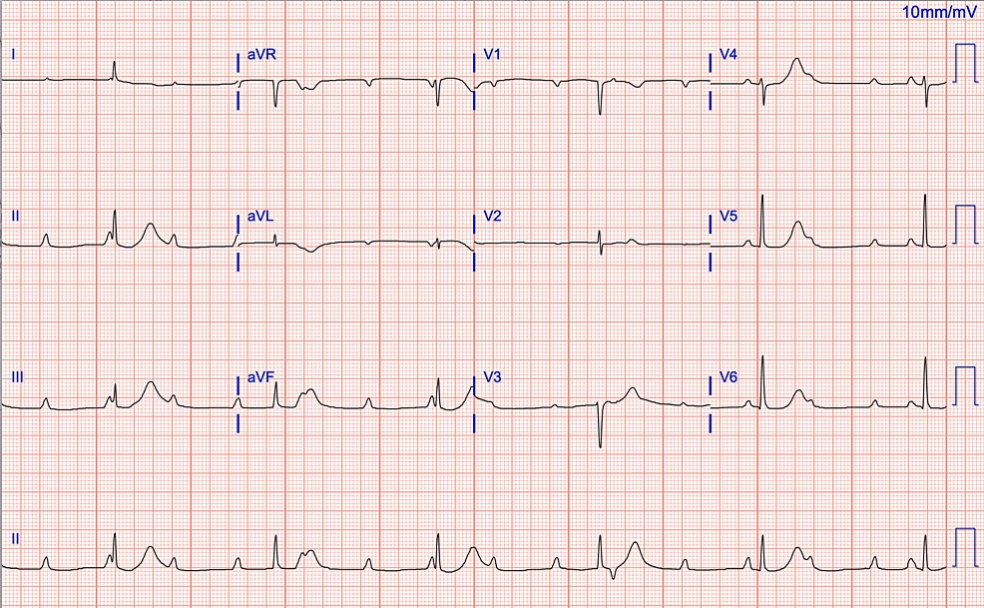
Complete heart block with junctional rhythm.

An electrocardiography (ECG) in the clinic demonstrated CHB with a junctional escape rhythm (Figure 1). She was sent to the emergency department due to the profound bradycardia. Given her symptoms suggestive of an upper respiratory tract infection, a multiplex real-time polymerase chain reaction was performed, testing positive for influenza A, while tests for COVID-19, influenza B, and respiratory syncytial virus were negative. A complete blood count revealed a white blood cell count of 4.6 x 10µ/uL with 41% neutrophils, 40.1% lymphocytes, normal red blood cell counts with macrocytosis, and a platelet count of 120 x 10^3^/µL (chronically low). Blood electrolytes showed sodium at 140 mmol/L, potassium at 3.7 mmol/L, and magnesium at 2 mg/dL, with normal renal functions. The thyroid-stimulating hormone was 1.55 µIU/mL with free T4 at 1.35 ng/dL. Lyme total antibody index was 0.09, which was within the normal range. Table 1 summarizes the patient’s laboratory findings. High-sensitivity troponin T had an increasing trend (23→50→53 ng/L); however, the one-hour and three-hour delta T were -3 and -4, respectively. In addition, subsequent ECGs did not show ischemic changes. An echocardiogram showed mildly increased left ventricular (LV) cavity size, with a left ventricular internal diameter of 4.2 cm and mildly concentric LV hypertrophy with an ejection fraction of 50-55%. There was no evidence of diastolic dysfunction or atrial enlargement.

**Table 1:** The patient’s laboratory evaluation. WBC: white blood cell count; RBC: red blood cell count; MCV: mean corpuscular volume; MCH: mean corpuscular hemoglobin; MCHC: mean corpuscular hemoglobin concentration; RDW-CV: red cell distribution width - coefficient of variation; BUN: blood urea nitrogen; ALT: alanine aminotransferase; AST: aspartate aminotransferase; TSH: thyroid-stimulating hormone

Lab	Results	Normal ranges
Complete blood count		
WBC	4.6 × 10^3^/µL	5.0–10.0 × 10^3^/µL
RBC	4.6 × 10^6^/µL	3.8–5.0 × 10^6^/µL
Hemoglobin	15.0 g/dL	12.0–15.0 g/dL
Hematocrit	47.5%	36–42%
MCV	103.7 fL	80–94 fL
MCH	32.8 pg	28–32 pg
MCHC	31.6 g/dL	32–36 g/dL
RDW-CV	12%	11.5–14.5 %
Platelets	120 × 10^3^/µL	150–400 × 10^3^/µL
Neutrophils	41.7%	50.0–70.0 %
Lymphocytes	40.1%	20–40.0 %
Monocytes	13.0%	0–10.0 %
Eosinophils	2.0%	0–5.0 %
Chemistry		
Sodium	140 mmol/L	136–145 mmol/L
Potassium	3.7 mmol/L	3.5–5.1 mmol/L
Chloride	102 mmol/L	98–107 mmol/L
Bicarbonate	25 mmol/L	21–32 mmol/L
Creatinine	0.87 mg/dL	0.60–1.00 mg/dL
BUN	13 mg/dL	7–18 mg/dL
Glucose	107 mg/dL	70–100 mg/dL
Calcium	9.4 mg/dL	8.5–10.5 mg/dL
Magnesium	2 mg/dL	1.8–2.5 mg/dL
Phosphorus	3.6 mg/dL	2.5–4.9 mg/dL
ALT	53 U/L	14–63 U/L
AST	52 U/L	10–42 U/L
Total protein	7.8 g/dL	6.0–8.0 g/dL
Albumin	3.9 g/dL	3.4–5.0 g/dL
High-sensitivity troponin T	23 ng/L	<14 ng/L
Other		
TSH	1.550 µIU/mL	0.270–4.200 µIU/mL
Free T4	1.35 ng/dL	0.80–1.70 ng/dL
Lyme antibody index	0.09	≤1.0

**Figure 2:**
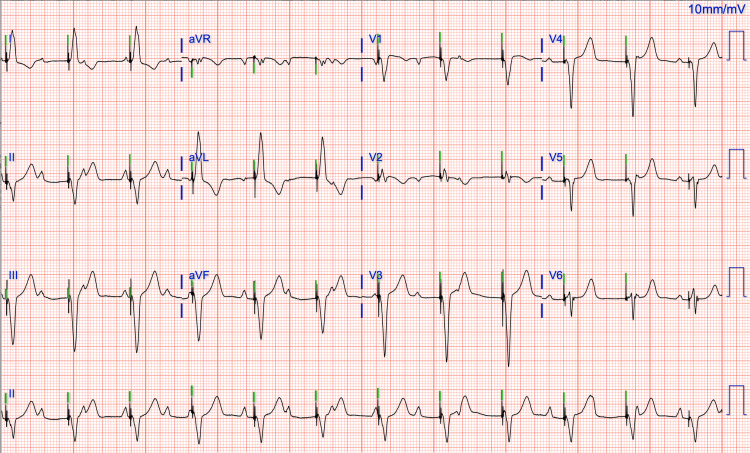
Post-permanent pacemaker shows the atrium sensed and the ventricle packed.

The patient was taken to the catheterization lab and had a transvenous temporary pacemaker inserted. As no resolution of heart block was noted, the patient underwent dual-chamber pacemaker implantation, in DDD mode, due to persistent CHB. Post-procedural ECG showed the atrium sensed and the ventricle paced (Figure 2). She was discharged with metoprolol due to elevated blood pressure with a high sinus rate. In her subsequent follow-up, she remained dependent on the pacemaker.

## Discussion

The cardiovascular implications of influenza are relatively underemphasized. Observational studies have shown a strong association between influenza and higher mortality rates among patients with cardiovascular disease. Furthermore, influenza has been associated with heart failure exacerbation and increased cardiovascular mortality. However, less attention has been given to the arrhythmogenic complications of influenza [13].

The initial correlation between influenza and cardiac rhythm abnormalities was noted in 1919 by Cockayne et al. [14], with the majority of cases including bradycardia and low-grade atrioventricular block. Subsequently, various arrhythmias proceeding with influenza infection have been reported, including atrial [15] and ventricular tachyarrhythmia [3,16], in addition to atrioventricular blocks [10].

Our patient’s presentation was rather nonspecific, characterized by flu-like symptoms without a significant cardiac history. Incidentally, a low heart rate was noted, different from her prior office readings, warranting an ECG evaluation that revealed CHB. While she tested positive for influenza A infection, her symptoms were minimal. Such presentation differs from prior reports of influenza-related atrioventricular block, which usually presents with sinus bradycardia or low-grade nodal atrioventricular block. While infra-nodal atrioventricular block has also been reported, it has been documented among patients with severe influenza infection and fatal myocarditis [7]. Beinart et al. [10] noted CHB following influenza A infection in a patient with global cardiac enhancement in cardiac imaging, suggesting underlying myocardial involvement. Such presentation has been observed to a greater extent among severe COVID-19 cases [17].

There is a scarcity of literature on the exact mechanism by which influenza exerts its effect on the heart. Vascular and myocardial injuries have been observed in numerous studies. Notably, heart rate and rhythm aberrations are believed to result from myocardial inflammation and viral replication, which promote myocarditis. In vitro studies have shown that the influenza virus can induce inflammation in cardiomyocytes, potentially leading to tissue fibrosis and apoptosis. These findings typically manifest clinically as reduced ejection fraction and acute heart failure. Nevertheless, in many cases, such as with our patient, manifestations of myocardial injury may be subtle, especially among patients with structurally preserved heart function [13]. In a review by Lippi et al. [18], a modest troponin elevation was observed in influenza infections, particularly influenza A, which is generally transient and self-limiting. Notably, our patient exhibited a slight elevation of high-sensitivity troponin, with no ischemic changes in the ECG. Furthermore, the echocardiography did not show evidence of diastolic dysfunction or wall motion abnormalities. We attribute this mild troponin elevation to possible mild myocardial inflammation, as similarly reported by Ergle et al. [11] and Cheng et al. [12]. In Table 2, we summarize previously documented influenza-induced CHB cases.

**Table 2:** Previous descriptions of complete heart block in influenza cases. AV: atrioventricular; CHB: complete heart block

Study	Patient characteristics	Rhythm abnormality	Associated condition	Intervention	Outcome
Beinart et al. [10]	An 18-year-old female, with a prior history of asthma	Initial intermittent 2:1 AV block, followed by CHB	Influenza pneumonia and respiratory failure. Possible myocarditis	Dual-chamber pacemaker	Complete resolution
Ergle et al. [11]	A 50-year-old female, with a prior history of hypothyroidism and depression	Initial CHB, followed by high-grade AV block	No myocarditis reported	Dual-chamber pacemaker	Persistent CHB
	A 20-year-old male, with a prior history of complicated congenital heart disease	Right and left bundle conduction disease and intermittent high-grade AV block	Sepsis and ITP. No myocarditis reported	Dual-chamber pacemaker	Complete resolution
Cheng et al. [12]	An 86-year-old female, with no significant medical history	CHB	No myocarditis reported	Dual-chamber pacemaker	Complete resolution
Gadela et al. [19]	A 58-year-old female with a history of first-degree AV block	Initial Mobitz type II, followed by CHB	FDG PET cardiac sarcoidosis (inactive). No myocarditis reported	Dual-chamber pacemaker	Not reported
Ukimura et al [20]	A 34-year-old female, with no significant medical history	CHB	Fulminant myocarditis	Hemodynamic and ventilatory support. Plasmapheresis	Death
	A 53-year-old male, with no significant medical history	CHB	No myocarditis reported	Temporary pacemaker	Resolution
	A 66-year-old male, with a prior history of emphysema	Ventricular fibrillation. CHB	No myocarditis reported	Ventilatory support	Resolution
	A 69-year-old male, with a prior history of emphysema and malignancy	CHB	Fulminant myocarditis	Mechanical and ventilatory support	Death

Although individuals with pre-existing heart conditions are assumed to be more susceptible to influenza complications, no distinct pattern was discernible from these case studies. Only a single case demonstrated a past medical history of a first-degree atrioventricular block, which transitioned into CHB after a flu infection [19]. In a national survey by Ukimura et al. [20] on the 2009 influenza pandemic, four cases of influenza-related CHB were reported. Fulminant myocarditis was observed in two patients, both of whom required ventilatory and hemodynamic support and demised during their illness. One case exhibited wall motion abnormalities and required a temporary pacemaker, which subsequently improved. This should highlight the variable spectrum of influenza-induced CHB, ranging from a mild upper respiratory tract infection to fulminant myocarditis.

Our patient was managed initially with temporary transvenous pacing, which unfortunately did not show improvement from CHB and resulted in the implantation of a permanent dual-chamber pacemaker. Noticeably, while most patients with influenza-induced CHB witnessed a full recovery [8,9,14], our patient continued to be dependent on the pacemaker. This is probably the second reported case of permanent CHB after influenza infection [11].

## Conclusions

Influenza virus can be associated with various forms of cardiac arrhythmia. In most patients, these arrhythmias are self-limiting and resolve completely. Complete atrioventricular block, although considerably rare, has been reported in a few cases in the literature that have been associated with severe myocarditis. We report a rare case of permanent CHB manifesting with mild respiratory symptoms. It is crucial to maintain vigilance to identify this potentially life-threatening arrhythmia. Management with a dual-chamber pacemaker is generally sufficient.
